# Supplementary figures and images for: Microbial diversity of the remote Trindade Island, Brazil: a systematic review
Source: PeerJ. 2025 Apr 30;13:e19305. doi: 10.7717/peerj.19305 (PMC12049103; doi:10.7717/peerj.19305)

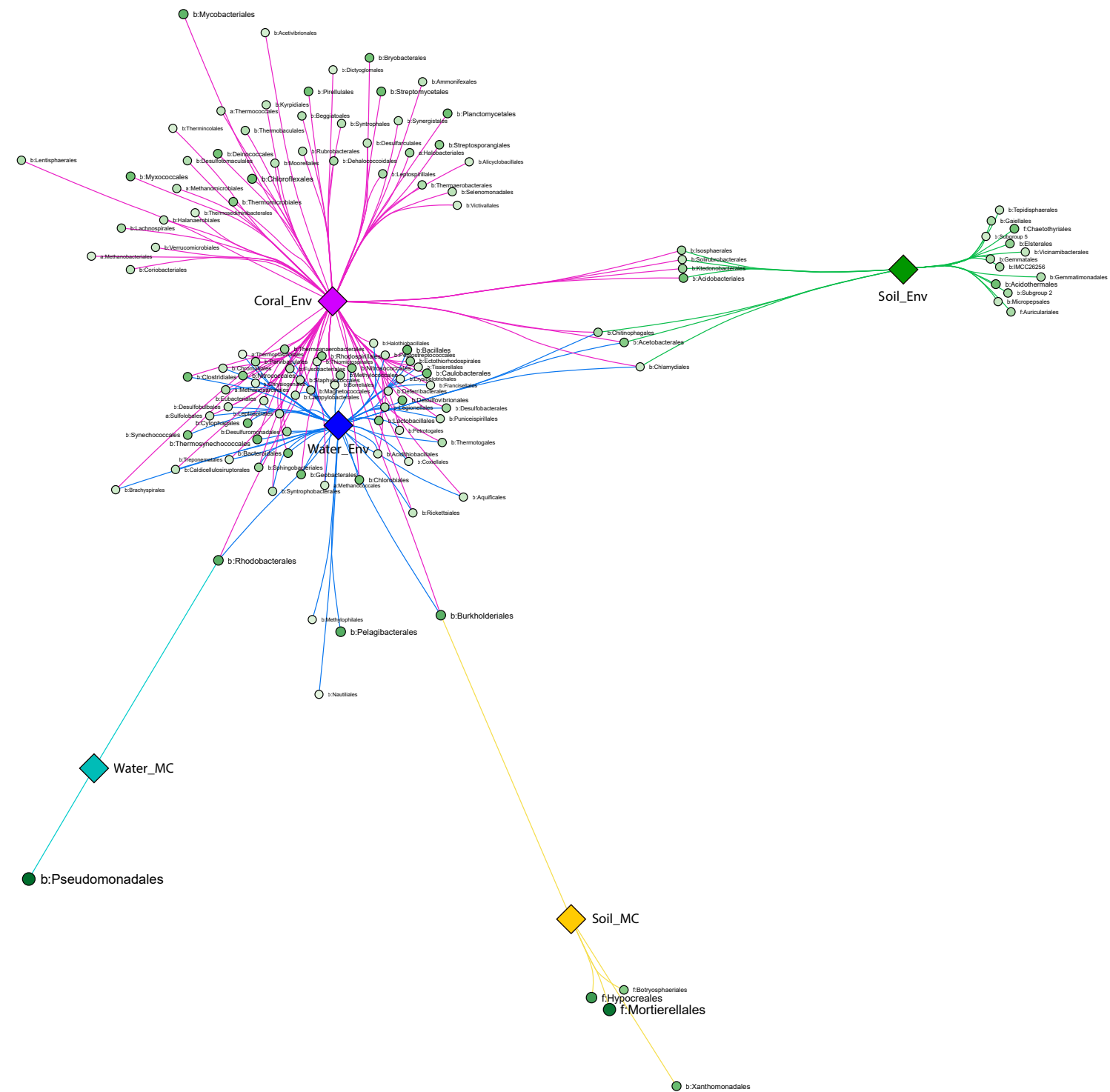

Supplement: Supplemental Information 1 [file peerj-13-19305-s001.pdf]

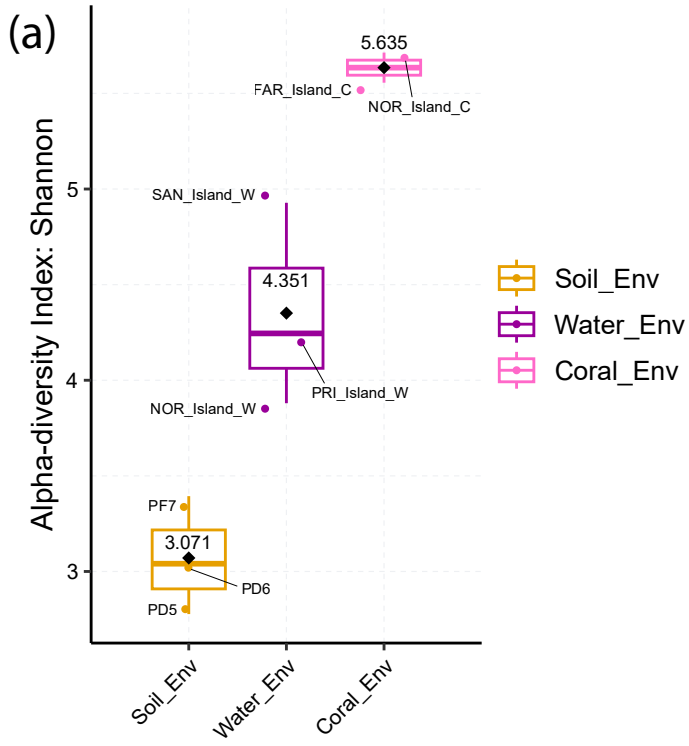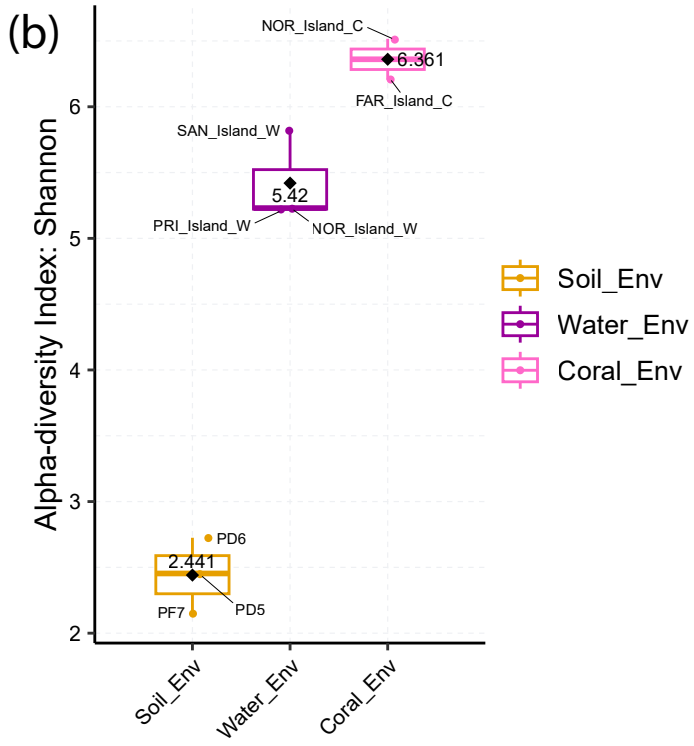

Supplement: Supplemental Information 2 [file peerj-13-19305-s002.pdf]
